# In-person versus virtual administration of the American College of Rheumatology gold standard cognitive battery in systemic lupus erythematosus: Are they interchangeable?

**DOI:** 10.1177/09612033231168477

**Published:** 2023-04-10

**Authors:** ML Barraclough, JP Diaz-Martinez, A Knight, K Bingham, J Su, M Kakvan, C Muñoz Grajales, MC Tartaglia, L Ruttan, J Wither, MY Choi, D Bonilla, N Anderson, S Appenzeller, B Parker, P Katz, D Beaton, R Green, IN Bruce, Z Touma

**Affiliations:** 1 Schroeder Arthritis Institute, Krembil Research Institute, 7989 University Health Network, Toronto, ON, Canada; 2 Centre for Epidemiology Versus Arthritis, Division of Musculoskeletal and Dermatological Sciences, School of Biological Sciences, Faculty of Biology, Medicine and Health, 5292 The University of Manchester, Manchester, UK; 3 NIHR Manchester Biomedical Research Centre, Manchester Academic Health Science Centre, Manchester University NHS Foundation Trust, Manchester, UK; 4 Centre for Prognosis Studies in Rheumatic Diseases, Toronto Western Hospital, 7938 University of Toronto Lupus Clinic, Toronto, ON, Canada; 5 Division of Rheumatology, 7979 Hospital for Sick Children, Toronto, ON, Canada; 6 Neurosciences and Mental Health Program, SickKids Research Institute, Toronto, ON, Canada; 7 Centre for Mental Health, 7989 University Health Network, Toronto, ON, Canada; 8 Department of Psychiatry, 7938 University of Toronto, Toronto, ON, Canada; 9 Krembil Research Institute, 7989 University Health Network Memory Clinic, Toronto, ON, Canada; 10 7961 University Health Network-Toronto Rehabilitation Institute, Toronto, ON, Canada; 11 Cumming School of Medicine, 70401 University of Calgary, Calgary, AB, Canada; 12 Department of Orthopaedics, Rheumatology and Traumatology, 7938 University of Campinas, São Paulo, Brazil; 13 8785 University of California, San Francisco, CA, USA; 14 Institute for Work and Health, 7966 University of Toronto, Toronto, ON, Canada

**Keywords:** systemic lupus erythematosus, neuropsychiatric lupus, cognitive impairment

## Abstract

**Objective:**

During the COVID-19 pandemic, many research studies were adapted, including our longitudinal study examining cognitive impairment (CI) in systemic lupus erythematosus (SLE). Cognitive testing was switched from in-person to virtual. This analysis aimed to determine if the administration method (in-person vs. virtual) of the ACR-neuropsychological battery (ACR-NB) affected participant cognitive performance and classification.

**Methods:**

Data from our multi-visit, SLE CI study included demographic, clinical, and psychiatric characteristics, and the modified ACR-NB. Three analyses were undertaken for cognitive performance: (1) all visits, (2) non-CI group visits only and (3) intra-individual comparisons. A retrospective preferences questionnaire was given to participants who completed the ACR-NB both in-person and virtually.

**Results:**

We analysed 328 SLE participants who had 801 visits (696 in-person and 105 virtual). Demographic, clinical, and psychiatric characteristics were comparable except for ethnicity, anxiety and disease-related damage. Across all three comparisons, six tests were consistently statistically significantly different. CI classification changed in 11/71 (15%) participants. 45% of participants preferred the virtual administration method and 33% preferred in-person.

**Conclusions:**

Of the 19 tests in the ACR-NB, we identified one or more problems with eight (42%) tests when moving from in-person to virtual administration. As the use of virtual cognitive testing will likely increase, these issues need to be addressed – potentially by validating a virtual version of the ACR-NB. Until then, caution must be taken when directly comparing virtual to in-person test results. If future studies use a mixed administration approach, this should be accounted for during analysis.

## Introduction

One of the effects of the COVID-19 pandemic was the suspension of many health research projects as face-to-face contact needed to be limited. As such, researchers tried to adapt their studies to use a virtual environment where possible.^1^ Virtually testing cognitive function is possible, and prior to the pandemic, there was already a shift to online assessment, especially using computerised cognitive tests.^2^

CANTAB® and ANAM are two established systems that provide validated normalised computerised cognitive batteries.^3^ Administration of these systems requires minimal training, and reporting is much easier as it automatically calculated. CANTAB® has an established online version of their tasks^4^ and ANAM has recently released an online version. CANTAB® studies have found many of the tests to be comparable in both in-person and online versions. However, caution must be used when assessing response time measures. These can be affected by both internet speed and hardware use, for example, mouse versus touch screen.^5^

The use of ANAM and CANTAB® to test cognitive impairment (CI) in systemic lupus erythematosus (SLE) is increasing but the American College of Rheumatology neuropsychological battery (ACR-NB) of tests remains the gold standard.^6^ This battery is primarily a face-to-face paper assessment. It includes 19 tests examining six different cognitive domains. Understanding how performance may be affected when this non-computerised assessment is administered in a virtual manner is currently unknown.

Prior to the COVID-19 pandemic, we had started a longitudinal project examining CI in SLE, with participant data collected at 0, 6, 12 and 24 months. Our protocol included the ACR-neuropsychological battery (ACR-NB) and ANAM, and all study visits were conducted face-to-face in-person. The study was suspended at the start of the pandemic in 2020 but parts were reopened including the use of an adapted version of the ACR-NB, which allowed virtual administration to start in February 2021.

The purpose of this study was to assess the comparability of the ACR-NB when administered in-person versus virtually. In addition, we explored which administration method participants preferred.

## Methods

Data from participants enrolled in our longitudinal CI in SLE study was used. This data included demographic, clinical, and psychiatric characteristics, and the modified American College of Rheumatology neuropsychological battery (ACR-NB), full details are published elsewhere.^3,7^ Participants in this study have up to four research visits at 0, 6, 12 and 24 months. Inclusion criteria required all participants to meet 2019 EULAR/ACR classification criteria for SLE,^8^ to be aged between 18 and 65 years old and to have an adequate level of English to enable completion of the cognitive tasks. Consecutive participants were approached from the Toronto Lupus Clinic at the University Health Network (UHN) Toronto Western Hospital. Patients provided written informed consent in accordance with the Helsinki Declaration and the study was reviewed and approved by the UHN Research Ethics Board (CAPCR ID: 15-9582).

In-person data was collected between July 2016 and 16^th^ March 2020. The study was then paused due to COVID-19 restrictions. Web-based virtual cognitive assessments began 8^th^ February 2021 and are still ongoing.

### Cognitive tests

#### In-person

The 1-h ACR-NB of tests were administered to participants by a psychometrist (Table 1). This battery includes 19 different tests split into six cognitive domains. One alteration to the ACR-NB was made, the Hopkins Verbal Learning Test-Revised^9^ was used instead of the California Verbal Learning Test.^10^

**Table 1.** Cognitive tests administered and the differences between the in-person and virtual tests.

Domain	In-person tests	Virtual tests
1. Manual motor speed and dexterity	1.1 dominant hand tapping	**Unable to do virtually**
	1.2 Non dominant hand tapping	
2. Simple attention and processing speed	2.1 Trails A^25^	**2.1 Trails A – changed from written to verbal**
	2.2 Stroop colour naming	2.2 Stroop colour naming
	2.3 Stroop word reading^26^	2.3 Stroop word reading
3. Visual-spatial construction	3.1 RCFT copy^27^	3.1 RCFT copy
4. Verbal fluency	4.1 COWAT	4.1 COWAT
	4.2 ANIMALS	4.2 ANIMALS
5. Learning and memory	Visuospatial memory^27^:	Visuospatial memory:
	5.1 RCFT recall	5.1 RCFT recall
	5.2 RCFT delay recall	5.2 RCFT delay recall
	5.3 RCFT recognition	5.3 RCFT recognition
	Verbal memory^9^:	Verbal memory:
	5.4 HVLT-R delayed recall	5.4 HVLT-R delayed recall
	5.5 HVLT-R recognition	5.5 HVLT-R recognition
	5.6 HVLT-R total recall	5.6 HVLT-R total recall
6. Executive function	6.1 Stroop interference score^26^	6.1 Stroop interference score
	6.2 WAIS letter number sequencing^28^	6.2 WAIS letter number sequencing
	6.3 WAIS-III digit symbol^29^	**6.3 SDMT (verbal version of WAIS-III digit symbol)**
	6.4 Trails B^25^	**6.4 Trails B – changed from written to verbal**
	6.5 Consonant trigrams^30^	6.5 Consonant trigrams

BOLD indicates significant changes to tests administered. COWAT: controlled oral word association test, RCFT: Rey complex figure test, HVLT-R: Hopkins verbal learning test-revised, WAIS: Wechsler adult intelligence scale, SDMT: symbol digit modalities test.

#### Virtual

The administration of the ACR-NB in a virtual environment was conducted in the same way as the in-person administration except that it was done through a video call. This also meant changes were required to five of the tests; two had to be completely removed and three were changed from written to verbal tests, see Table 1.

All cognitive test scores were converted into z-scores.

After completion of the battery, participants’ level of cognitive function was determined. Participants were categorised as cognitively impaired if they met the following criteria (CI algorithm):

Participants must have impaired performance in two or more domains. Domains 1–4 were considered impaired if one or more tests within the domains had z-score ≤ −1.5. Domains 5 and required two or more tests to have z-score ≤ −1.5.^11^

Participants were further categorised into one of three groups based on their cognitive functioning over time. These groups were defined as follows:

1. No CI, participant did not meet the definition for CI at any study visit.
2. Fluctuating CI, participant was impaired at some visits and not at others.
3. Persistent CI, participant was impaired at all study visits.

An additional questionnaire was added retrospectively to ask participants about their experiences of in-person versus virtual visits. This questionnaire was given to all participants that undertook both virtual and in-person cognitive assessments (Supplementary Figure 1).

#### Analysis

All data available was assessed for completeness. Visits with ≥5 cognitive test results or ≥2 cognitive domains missing were removed from the analysis. Data was split into two groups, in-person versus virtual visits. Characteristics were compared between in-person and virtual visit data using Mann–Whitney U or chi-square.

Three different analyses were conducted on the cognitive data to examine differences between in-person and virtual assessment performance, using Mann–Whitney U or Wilcoxon Signed Ranks test:

1. Analysis of all available visit data.
2. Analysis of visit data from participants who remained non-cognitively impaired over all visits.
3. Intra-individual longitudinal analysis of those who had both an in-person and virtual visit at different time points. An additional delta analysis was also performed. This examined change in performance from two in-person visits compared to change in performance from an in-person visit and virtual visit.

The purpose of the three analyses enabled us to control for potentially confounding factors. Analysis 1 provided an overview of all data available. Analysis 2 allowed us to control for participants who may have naturally fluctuating CI levels over time. Analysis 3 enabled us to control for other factors that may affect cognition, such as medication, depression and disease damage, but may remain more stable within participants.

## Results

Results from 816 visits were available. After removing visits with missing data, 801 visits were included. 328 participants contributed to these visits. In-person visits accounted for 696 visits and virtual 105 visits. Baseline characteristics, split by administration method can be seen in Supplementary Table 1. No significant differences were seen in visit characteristics except for ethnicity, disease damage and anxiety, where higher level of damage was noted amongst the in-person visits and higher level of anxiety amongst the virtual visits. In terms of ethnicity, there were more Black and Caucasian in-person visits compared to more Asian and Other ethnicities for the virtual visits.

Across both visit types, disease activity (as assessed by the SLEDAI) was seen in the following systems: central nervous *n* = 15 (1.8%), vascular *n* = 14 (1.7%), musculoskeletal *n* = 54 (6.7%), renal *n* = 96 (12%), dermatologic *n* = 103 (13%), serosal *n* = 10 (1.2%), immunologic *n* = 467 (58%), constitutional *n* = 10 (1.2%) and haematologic *n* = 63 (7.8%). Also, across both visits types, 300 visits had patients with antiphospholipid syndrome and ACR criteria categories were split as follows: malar rash *n* = 543 (68%), discoid rash n = 111 (14%), photosensitivity *n* = 467 (58%), oral ulcers *n* = 432 (54%), arthritis *n* = 658 (82%), serositis *n* = 263 (33%), renal disorder *n* = 345 (43%), neurologic disorder *n* = 75 (9%), haematologic disorder *n* = 707 (88%), immunologic disorder *n* = 726 (91%) and antinuclear antibody positive n = 795 (99%).

### All visits

Differences were found between in-person and virtual visits for 10/17 cognitive tests in the domains of simple attention and processing speed, learning and memory, and executive function. Performance at virtual visits compared to in-person were improved for Trails A and B, Rey Complex Figure Test (RCFT), Stroop interference score and Auditory consonant trigrams. Performance at virtual visits compared to in-person worsened on Stroop colour naming and word reading and Symbol Digit Modalities Test (SDMT) compared to WAIS-III digit symbol (Table 2).

**Table 2.** Comparisons between in-person and virtual visits cognitive test results for *all visits*.

	In-person (*n* = 696)	Virtual (*n* = 105)	*p*-value
	Median z-scores (LQ, UQ)	Median z-scores (LQ, UQ)	
**2.1 Trails A**	**0.73 (−0.04, 1.19)**	**2.13 (1.86, 2.50)**	**<0.001**
**2.2 Stroop colour naming**	**0.04 (−0.67, 0.88)**	**−0.47 (−1.17, 0.38)**	**0.001**
**2.3 Stroop word reading**	**−0.41 (−1.13, 0.41)**	**−1.13 (−2.05, −0.41)**	**<0.001**
3 RCFT copy	−0.59 (−2.34, 0.12)	−0.95 (−2.78, −0.01)	0.122
4.1 COWAT	−0.24 (−0.96, 0.47)	−0.33 (−1.15, 0.47)	0.164
4.2 ANIMALS	0.20 (−0.54, 1.00)	0.20 (−0.53, 0.85)	0.926
**5.1 RCFT recall**	**−0.39 (−1.38, 0.60)**	**−0.14 (−1.10, 1.00)**	**0.047**
**5.2 RCFT delay recall**	**−0.28 (−1.41, 0.61)**	**0.20 (−1.08, 0.81)**	**0.046**
**5.3 RCFT recognition**	**−0.28 (−0.99, 0.28)**	**0.08 (−0.81, 0.99)**	**0.004**
5.4 HVLT-R delayed recall	−0.61 (−1.75, 0.28)	−0.18 (−1.78, 0.41)	0.224
5.5 HVLT-R recognition	−0.52 (−2.05, 0.47)	−0.75 (−1.67, 0.50)	0.894
5.6 HVLT-R total recall	−0.81 (−1.48, 0.00)	−0.65 (−1.53, 0.05)	0.696
**6.1 Stroop interference score**	**0.61 (−0.08, 1.34)**	**0.88 (0.26, 1.48)**	**0.001**
6.2 WAIS letter number sequencing	0.00 (−0.67, 0.67)	0.00 (−0.67, 0.67)	0.505
**6.3 WAIS-III digit symbol/SDMT**	**0.33 (−0.33, 0.99)**	**−0.80 (−1.40, −0.13)**	**<0.001**
**6.4 Trails B**	**0.53 (−0.59, 1.30)**	**1.86 (0.88, 2.34)**	**<0.001**
**6.5 auditory consonant trigrams test**	**−0.80 (−1.61, 0.16)**	**−0.35 (−1.31, 0.40)**	**0.008**

RCFT: rey complex figure test, COWAT: controlled oral word association test, HVLT-R: Hopkins verbal learning test-revised, WAIS: Wechsler adult intelligence scale, SDMT: symbol digit modalities test. Bold = statistically significant.

#### Visits of those with no CI

A total of 394 visits were classified as not cognitively impaired, comprised of 346 in-person visits and 48 virtual visits.

Seven out of 17 tests were significantly different between non-CI virtual and in-person visits. Similar to all visits, improved performance was seen in Trails A and B, RCFT and Stroop interference score for virtual versus in-person visits. Poorer performance was seen on the Stroop colour naming and word reading and SDMT compared to WAIS-III digit symbol for virtual versus in-person visits (Table 3).

**Table 3.** Comparisons between in-person and virtual visits cognitive test results for *non-CI visits only*.

	In-person (*n* = 346)	Virtual (*n* = 48)	*p*-value
	Median z-scores (LQ, UQ)	Median z-scores (LQ, UQ)	
**2.1 Trails A**	**0.97 (0.38, 1.44)**	**2.13 (1.96, 2.46)**	**<0.001**
**2.2 Stroop colour naming**	**0.61 (−0.20, 1.17)**	**0.08 (−0.81, 0.67)**	**0.003**
**2.3 Stroop word reading**	**0.00 (−0.61, 0.67)**	**−0.61 (−1.48, 0.00)**	**<0.001**
3 RCFT copy	−0.15 (−0.75, 0.43)	−0.26 (−1.09, 0.44)	0.596
4.1 COWAT	0.08 (−0.60, 0.69)	−0.19 (−1.04, 0.55)	0.125
4.2 ANIMALS	0.39 (−0.29, 1.13)	0.76 (0.06, 1.13)	0.391
5.1 RCFT recall	0.35 (−0.48, 1.15)	0.65 (−0.10, 1.43)	0.085
5.2 RCFT delay recall	0.41 (−0.52, 1.04)	0.44 (0.00, 1.17)	0.304
**5.3 RCFT recognition**	**−0.20 (−0.81, 0.47)**	**0.35 (−0.46, 1.15)**	**0.010**
5.4 HVLT-R delayed recall	0.00 (−0.99, 0.47)	0.25 (−0.76, 0.82)	0.100
5.5 HVLT-R recognition	0.41 (−0.92, 0.47)	0.33 (−0.75, 0.50)	0.390
5.6 HVLT-R total recall	−0.28 (−1.00, −0.28)	−0.38 (−0.74, 0.15)	0.996
**6.1 Stroop interference score**	**0.81 (0.08, 1.64)**	**1.38 (0.61, 2.33)**	**0.005**
6.2 WAIS letter number sequencing	0.33 (−0.33, 0.67)	0.00 (−0.59, 0.99)	0.509
**6.3 WAIS-III digit symbol/SDMT**	**0.67 (0.00, 1.34)**	**0.28 (−0.77, 0.30)**	**<0.001**
**6.4 Trails B**	**1.00 (0.22, 1.65)**	**2.14 (1.37, 2.40)**	**<0.001**
6.5 auditory consonant trigrams test	−0.30 (−1.20, 0.58)	−0.09 (−0.75, 0.71)	0.099

RCFT: Rey Complex Figure Test, COWAT: Controlled Oral Word Association Test, HVLT-R: Hopkins Verbal Learning Test-Revised, WAIS: Wechsler Adult Intelligence Scale, SDMT: Symbol Digit Modalities Test. Bold = statistically significant.

#### Intra-individual longitudinal comparisons

71 participants had at least one virtual and one in-person visit completed at different times. The mean time between visits was 2.15 (±0.98) years. Differences between these visits were seen in 7/17 of the cognitive tasks. Scores on Trails A and B, Stroop interference and auditory consonant trigrams were improved at the virtual compared to in-person visit. Stroop colour naming and word reading and SDMT compared to WAIS-III digit symbol scores were worse at the virtual compared to in-person visit (Table 4). The delta analysis included 60 participants and found significant differences for Trails A and B, Stroop interference, Stroop colour naming and word reading, and SDMT compared to WAIS-III digit symbol scores. The WAIS letter number sequencing also had a significant change in score (Supplementary Table 2).

**Table 4.** Intra-individual participant comparisons between in-person and virtual visits cognitive test results.

	In-person (*n* = 71)	Virtual (*n* = 71)	*p*-value
	Median z-scores (LQ, UQ)	Median z-scores (LQ, UQ)	
**2.1 Trails A**	**0.73 (0.16, 1.29)**	**2.23 (1.94, 2.54)**	**<0.001**
**2.2 Stroop colour naming**	**0.47 (−0.67, 1.17)**	**−0.47 (−1.17, 0.26)**	**<0.001**
**2.3 Stroop word reading**	**−0.35 (−1.10, 0.58)**	**−1.13 (−1.88, −0.41)**	**<0.001**
3 RCFT copy	−0.54 (−2.50, 0.04)	−0.95 (−2.86, −0.12)	0.214
4.1 COWAT	−0.34 (−1.07, 0.53)	−0.25 (−1.13, 0.51)	0.295
4.2 ANIMALS	0.20 (−0.54, 0.99)	0.29 (−0.29, 1.13)	0.526
5.1 RCFT recall	−0.18 (−1.48, 0.79)	0.08 (−1.11, 1.25)	0.191
5.2 RCFT delay recall	−0.08 (−1.17, 0.88)	0.28 (−1.34, 0.99)	0.064
**5.3 RCFT recognition**	**−0.28 (−1.17, 0.31)**	**0.28 (−0.41, 1.08)**	**0.017**
5.4 HVLT-R delayed recall	−0.52 (−1.75, 0.41)	−0.18 (−1.94, 0.41)	0.350
5.5 HVLT-R recognition	−0.67 (−2.05, 0.47)	−0.75 (−2.00, 0.50)	0.364
5.6 HVLT-R total recall	−0.74 (−1.75, 0.08)	−0.74 (−1.73, −0.21)	0.131
6.1 Stroop interference score	0.98 (0.22, 1.88)	0.88 (0.43, 1.85)	0.667
6.2 WAIS letter number sequencing	0.00 (−0.67, 0.67)	0.00 (−0.67, 0.67)	0.093
**6.3 WAIS-III digit symbol/SDMT**	**0.67 (0.00, 0.99)**	**−0.82 (−1.39, −0.01)**	**<0.001**
**6.4 Trails B**	**0.69 (−0.45, 1.39)**	**2.06 (0.86, 2.40)**	**<0.001**
**6.5 auditory consonant trigrams test**	**−0.71 (−1.66, 0.40)**	**−0.40 (−1.13, 0.40)**	**0.003**

RCFT: Rey Complex Figure Test, COWAT: Controlled Oral Word Association Test, HVLT-R: Hopkins Verbal Learning Test-Revised, WAIS: Wechsler Adult Intelligence Scale, SDMT: Symbol Digit Modalities Test. Bold = statistically significant.

The six tests that were consistently statistically different across all three analyses were Trails A (2.1), Stroop colour naming (2.2) and word reading (2.3), RCFT recognition (5.3), WAIS-III digit symbol/SDMT (6.3) and Trails B (6.4). Table 5 shows a summary of the statistical differences and directions of change for the cognitive tests across all three analyses. Supplementary Figure 2 illustrates the differences in cognitive performance between the in-person and virtual administration methods for each cognitive test across each analysis. For example, the all visits diagram shows the median performance on cognitive test 2.1 (Trails A) was better at the virtual compared to in-person visits. Whereas the performance on test 4.2 (ANIMALS) was very similar for both administration methods. These diagrams show very similar patterns, across the cognitive tests, for all three analyses and further highlight that tests 2.1, 2.2, 2.3, 5.3, 6.3 and 6.4 are the most affected by administration method.

**Table 5.** A summary of which cognitive tasks were significantly different for the three analyses.

Cognitive task	Virtual visits median z-score compared to in-person median z-score		
	All visits *n* = 801	Non-CI visits only *n* = 394	Intra-individual participants*n* = 71
2.1: Trails A*	**↑**	**↑**	**↑**
2.2: Stroop colour naming*	**↓**	**↓**	**↓**
2.3: Stroop word reading*	**↓**	**↓**	**↓**
5.1 RCFT recall	↑	ns	ns
5.2 RCFT delay recall	↑	ns	ns
5.3 RCFT recognition*	**↑**	**↑**	**↑**
6.1: Stroop interference score	↑	↑	ns
6.3: WAIS-III digit symbol (replaced by SDMT)*	**↓**	**↓**	**↓**
6.4: Trails B*	**↑**	**↑**	**↑**
6.5 auditory consonant trigrams	↑	ns	↑

*Statistically significant across all three analyses ↑Better performance, ↓Worse performance RCFT: Rey Complex Figure Test, WAIS: Wechsler Adult Intelligence Scale, SDMT: Symbol Digit Modalities Test.

#### Changes in CI status

**Figure 1.**
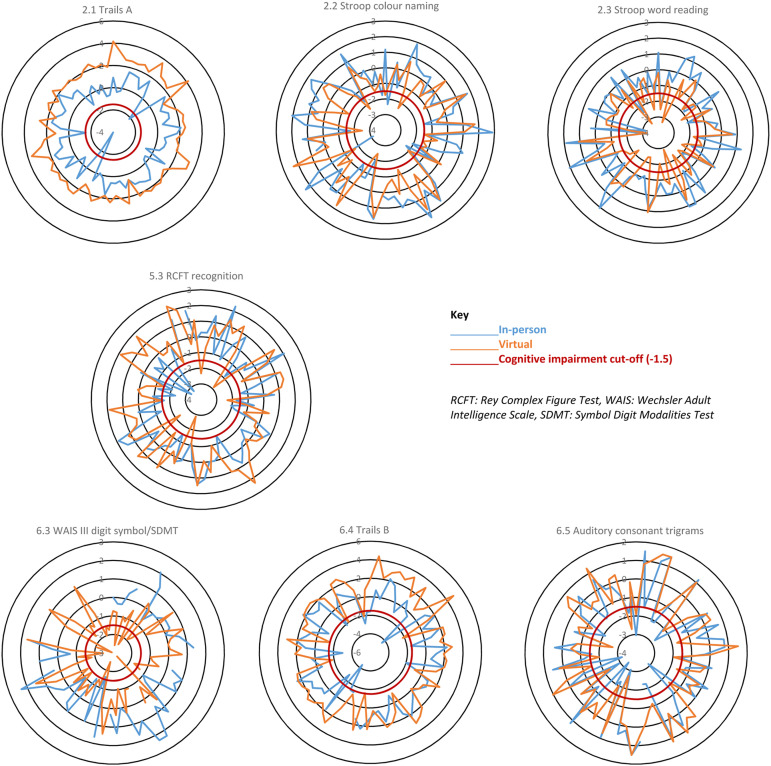
Spider diagrams of individual participant z-scores for the seven cognitive tests affected by administration method in the intra-individual analysis. These diagrams show results from the 71 participants who had both an in-person and virtual visit. Where only one result (in-person or virtual) crosses the red line this may indicate a change in CI status for that individual.

The intra-individual comparisons found that performance on seven of the cognitive tests were significantly different between virtual and in-person assessments. Further examination of how these differences may have affected participant CI status stratification, based on our CI algorithm, found that 11/71 (15%) participants had a change in CI status. Three participants were moved from CI into the not cognitively impaired group and eight from non-impaired into the impaired group. Figure 1 illustrates which participants’ test scores cross the −1.5 CI cut-off. If participant in-person and virtual scores are separated by the cut-off, this may have changed the participants CI status.

#### Preference questionnaire

Sixty-six out of 71 participants completed the preference questionnaire. The results showed a preference for the virtual assessment (*n* = 30, 45%), compared to *n* = 22, 33% for in-person and *n* = 14, 21% who had no preference. The main reason people preferred the virtual visit was that they did not need to travel; this was followed by ‘it is more convenient’ and ‘reduces in-person contact during COVID-19’. With regards to how the test is run, the majority of participants (*n* = 50, 76%) felt that there was no difference in difficulty between virtual and in-person testing. When asked if anything could make the virtual experience better, of the 61 who commented, the majority (*n* = 52) said they had no suggestions, five participants expressed a wish for more technical support and two participants suggested more information was needed regarding what additional equipment (e.g. pencils) they would need during the assessment.

## Discussion

Of the 19 tests in the ACR-NB, we identified one or more problems with eight (42%) tests as we moved from in-person to virtual administration including tests that cannot be administered virtually (*n* = 2), tests that required modification (*n* = 3), and tests (*n* = 6) that showed significant and consistent performance differences according to mode of administration. The tests primarily affected were those that had been changed from a written to verbal response and had a timed element as well as those that required a visual presentation. Three tasks, Trails A and B and WAIS-III digit symbol/SDMT, were changed from a written to verbal response. This change made both Trails A and B easier. Trails A is particularly affected when converted to verbal and other researchers have suggested that a verbal version of Trails A is not appropriate.^12^ In a similar remote study, in patients with multiple sclerosis, the Trail Making Tests were also used but to avoid issues with verbal versus written response, paper copies of the tests were posted out prior to the assessment to ensure that the test could be done in written form. The results were more comparable but participants still performed better on Trails A when administered remotely.^13^ Using a written version remotely may be a better method for ensuring comparability to in-person testing but is not ideal for the long-term switch to virtual assessment. If testing is to move to a remote setting long-term, then better computerised adaptations of the Trails Making Test are required.

A verbal version of the WAIS-III digit symbol test was also required for our study. As such, the WAIS-III digit symbol was replaced by the SDMT. These tests have been previously shown to be comparable,^14^ with the exception that the SDMT can be responded to verbally and not just in written form.^15^ For these tests, the participant is scored based on how many correct answers they can give within a set timeframe. The WAIS-III digit symbol test is assessed over 120 s, whereas the SDMT is shorter and assessed over 90 s, as such accounting for the speed advantage from a verbal response. However, our cohort performed worse on the SDMT in the virtual environment compared to the in-person administration of the WAIS-III digit symbol. This suggests additional adaptations are required to ensure that these tests are comparable in an SLE cohort; however, it is also worth noting that this task had some missing data.

The remaining three tests that were consistently significantly different were the Stroop colour naming and word reading and the RCFT recognition. For both Stroop results, participants performed worse during the virtual administration of this test compared to in-person. The reasoning for this altered performance is believed to be linked to the presentation of the stimuli. As with the in-person administration, the participant is presented with one sheet showing all the words they are required to read. In-person, they are handed a *letter size* sheet of paper. Virtually, all the words are presented onscreen. This latter version means the words are smaller and harder to read and therefore negatively affects performance. Other studies adapting cognitive tests from in-person to virtual have found similar issues due to display restrictions.^13^ Contrary to this, participants performed better on the RCFT recognition, this may be due to environment. For example, studies have shown cognitive performance is affected by stress and anxiety,^16,17^ states that can be affected by environment such as a home setting versus a laboratory setting.

The intra-individual longitudinal comparison found that seven tests were potentially affected by administration method. Further exploration of performance on these tests found that only 15% of the participants changed CI status (based on our CI algorithm) due to performance on one or more of these seven tests. This suggests that while there were statistically different results from virtual versus in-person cognitive testing, this may not result in a difference in CI classification.

This work was needed in response to adaptations made to our CI in SLE study because of the COVID-19 pandemic. As such, the analysis for this study was planned retrospectively and the ideal comparison of administering both methods (virtual and in-person) on the same day to the same participant in a counterbalanced way was not possible and must be considered a limitation of this work. Instead, we undertook three analyses to try and control for as many confounding factors as possible. Also, we were unable to counterbalance the administration method. All in-person visits were undertaken prior to the virtual visits, so we cannot rule out learned effects. It is also worth noting that the anxiety levels in the virtual administration method were higher than in-person. This difference is likely connected with the COVID-19 pandemic where global levels of anxiety increased.^18^ Finally, using computer technology in a study may have health inequality implications as it requires access to computers and the internet as well as computer literacy^19^ and therefore may have biased our study cohort or affected our results.

Assessing cognitive function virtually, especially for research, is likely to become more common even as we see COVID-19 restrictions lifted. Our research found that virtual administration was preferred over in-person testing by participants and there are many benefits for researchers too, mainly regarding time and flexibility to deliver the testing. As such, research needs to ensure that the newly adapted virtual tests are validated in the same way the in-person versions were. New cut-offs may need to be established to ensure comparability between the administration methods.^20–22^ Our study has shown that it is possible to adapt the ACR-NB but that caution needs to be taken if comparing in-person to virtual administration. Future research may find results more comparable if established computerised cognitive batteries, such as the CANTAB® and ANAM, are used instead.^23^ Ultimately, the new virtual way of working is likely to remain^24^ and future cognitive research needs to ensure accurate testing is available and validated.

## Supplemental Material

Supplemental Material - In-person versus virtual administration of the American College of Rheumatology gold standard cognitive battery in systemic lupus erythematosus: Are they interchangeable? Supplemental Material for In-person versus virtual administration of the American College of Radiology gold standard cognitive battery in systemic lupus erythematosus: Are they interchangeable? by ML Barraclough, JP Diaz-Martinez, A Knight, K Bingham, J Su, M Kakvan, C Muñoz Grajales, MC Tartaglia, L Ruttan, J Wither, MY Choi, D Bonilla, N Anderson, S Appenzeller, B Parker, P Katz, D Beaton, R Green, IN Bruce and Z Touma in Lupus
