# Supplementary material for: In-person versus virtual administration of the American College of Rheumatology gold standard cognitive battery in systemic lupus erythematosus: Are they interchangeable?
Source: Lupus. 2023 Apr 10;32(6):737–45. doi: 10.1177/09612033231168477 (PMC10189815; doi:10.1177/09612033231168477)
Supplement: Supplemental Material - In-person versus virtual administration of the American College of Rheumatology gold standard cognitive battery in systemic lupus erythematosus: Are they interchangeable? [file sj-pdf-1-lup-10.1177_09612033231168477.pdf]

Supplementary Table 1. Comparison of characteristics between the two administration methods (in-person vs virtual)

Variable	In-person (n=696) Median (LQ, UQ), n (%)	Virtual (n=105)	p-value
Age at assessment	41 (31, 52)	39 (31, 53)	0.913
Sex (female)	618 (89%)	97 (92%)	0.268
Age at diagnosis (years)	25 (19, 33)	25 (19, 33)	0.794
Disease duration at assessment (years)	13 (6, 22)	12 (4, 23)	0.567
Education:			0.434
• < grade 8	0	0	
• Grade 8	23 (3%)	2 (2%)	
• High school graduate	119 (17%)	17 (16%)	
• College	258 (37%)	33 (31%)	
• University	296 (43%)	53 (51%)	
**Ethnicity:**			**0.009**
• Caucasian	391 (57%)	45 (46%)	
• Black	140 (20%)	15 (15%)	
• Asian	70 (10%)	19 (20%)	
• Other	87 (13%)	18 (19%)	
SLEDAI-2K score	2 (0, 4)	2 (0, 4)	0.200
**SDI score**	**1 (0, 2)**	**0 (0, 1)**	**0.018**
Immunosuppressant use (y)	401 (58%)	71 (68%)	0.052
Current antimalarial use (y)	575 (83%)	82 (78%)	0.261
Glucocorticoid use (y)	326 (47%)	42 (40%)	0.190
Biologic medication use (y)	136 (20%)	14 (13%)	0.129
Beck Depression Inventory II	12 (6, 22)	13 (6, 26)	0.153
**Beck Anxiety Inventory**	**12 (6, 23)**	**34 (30, 48)**	**<0.001**
Cognitively impaired* (y)	241 (35%)	40 (38%)	0.488

SLEDAI-2K: Systemic Lupus Erythematosus Disease Activity Index-2000, SDI: Systemic Lupus International Collaborating Clinics/American College of Rheumatology Damage Index

**As per our algorithm*

For Peer Review

1. Having an option, which type of assessment do you prefer?
   a) In-person
   b) Virtual (online, remote)
   c) No preference
2. If you prefer
   a) In-person, why do you prefer this method?
      i. Easier to understand the questions
      ii. Do not need to use technology
      iii. Less stressful environment, more comfortable
      iv. It is more convenient
      v. Other…
   b) Virtual, why do you prefer this method?
      i. Easier to understand the questions
      ii. Less stressful environment, more comfortable
      iii. It is more convenient
      iv. Reduces in-person contact during COVID-19
      v. No need to travel
      vi. Other…
3. In terms of how the test is run, how would you rate virtual to in-person?
      i. Much easier
      ii. A little easier
      iii. Easier
      iv. No difference
      v. Harder
      vi. A lot harder
4. Is there anything that would make the virtual assessment a better experience?

Supplementary Figure 1. Preference questionnaire administered to study participants who had completed both virtual and in-person cognitive assessments.

Supplementary Table 2: Intra-individual participant delta differences between two in-person visits and one in-person and virtual visit

Cognitive test	Change in z-scores (median, LQ, UQ)		p-value
	In-person v1 minus in-person v2*	In-person v2 minus virtual v1^	
**2.1 Trails A**	**0.11 (-0.10, 0.46)**	**1.45 (1.01, 2.53)**	**<0.0001**
**2.2 Stroop colour naming**	**0.02 (-0.27, 0.63)**	**-0.58 (-1.21, -0.11)**	**<0.001**
**2.3 Stroop word reading**	**0 (-0.35, 0.45)**	**-0.59 (-1.24, -0.14)**	**<0.001**
3 RCFT copy	0 (-0.79, 0.79)	0 (-1.64, 0.87)	0.258
4.1 COWAT	0 (-0.40, 0.47)	0 (-0.71, 0.36)	0.305
4.2 ANIMALS	0 (-0.79, 0.74)	0.19 (-0.56, 0.56)	0.810
5.1 RCFT recall	0.24 (-0.24, 1.18)	0 (-0.51, 0.73)	0.143
5.2 RCFT delay recall	0.17 (-0.29, 1.10)	0.11 (-0.52, 0.87)	0.450
5.3 RCFT recognition	0 (-0.61, 1.03)	0.58 (-0.60, 1.33)	0.525
5.4 HVLT-R delayed recall	0 (-0.49, 0.58)	-0.03 (-1.17, 0.59)	0.125
5.5 HVLT-R recognition	0 (-0.55, 0.55)	0 (-1.25, 1.07)	0.487
5.6 HVLT-R total recall	0 (-0.44, 0.63)	-0.25 (-1.02, 0.53)	0.136
**6.1 Stroop interference score**	**0.40 (-0.26, 0.87)**	**0 (-0.54, 0.53)**	**0.013**
**6.2 WAIS letter number sequencing**	**0 (-0.34, 0.67)**	**0 (-0.67, 0.33)**	**0.04**
**6.3 WAIS-III digit symbol/SDMT**	**0.30 (-0.32, 0.66)**	**-1.05 (-1.86, -0.47)**	**<0.001**
**6.4 Trails B**	**0.16 (-0.35, 0.65)**	**1.04 (0.24, 2.27)**	**0.025**
6.5 Auditory consonant trigrams test	-0.04 (-0.76, 0.73)	0.24 (-0.31, 0.90)	0.121

**In-person v2 = scores from participants' most recent in-person visit, v1 = the closest visit to v2.*
^Virtual v1 = the participants' first virtual visit
COWAT: Controlled Oral Word Association Test, RCFT: Rey Complex Figure Test, HVLT-R: Hopkins Verbal Learning Test-Revised, WAIS: Wechsler Adult Intelligence Scale, SDMT: Symbol Digit Modalities Test

For Peer Review

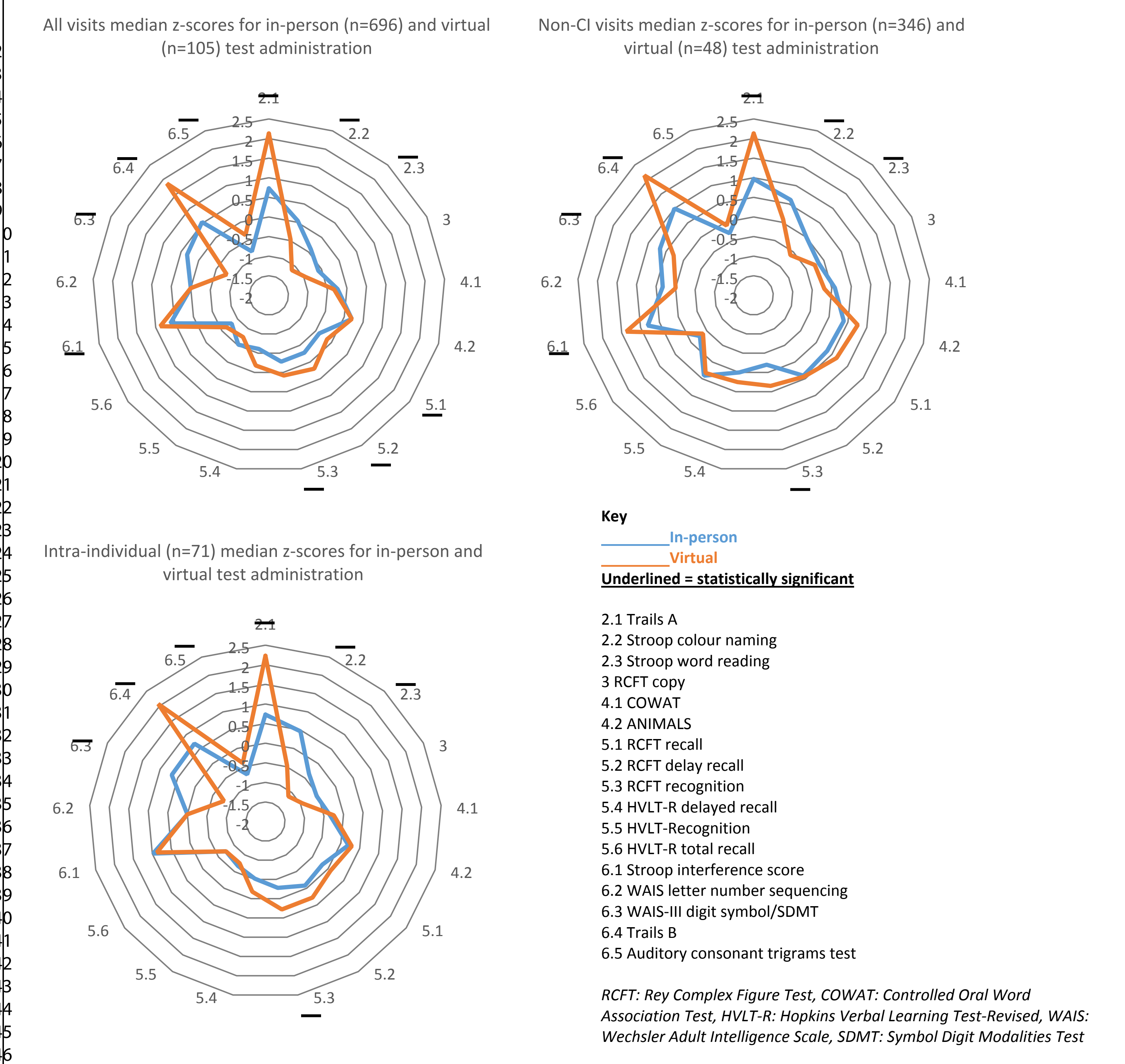

Supplementary Figure 2. Spider diagrams showing the differences in median cognitive performance z-scores between in-person and virtual administration methods for all cognitive tests, across all three analyses (all visits, non-CI visits only, intra-individual comparison).

These diagrams show very similar patterns for all three analyses and further highlight that tests 2.1, 2.3, 6.3 and 6.4 are the most affected by administration method.
